# Does acupuncture improve the metabolic outcomes of obese/overweight children and adolescents?: A systematic review and meta-analysis

**DOI:** 10.1097/MD.0000000000034943

**Published:** 2023-10-06

**Authors:** Tingwei Quan, Qi Su, Yu Luo, Xin Su, Qiuxuan Chen, Jingjun Yang, Hongzhen Tang

**Affiliations:** a Department of Acupuncture and Tuina, Guangxi University of Chinese Medicine, Nanning, Guangxi Province, China; b The First Affiliated Hospital of Guangxi University of Chinese Medicine, Nanning, Guangxi Province, China; c Wanfa Tang Traditional Chinese Medicine Clinic, Hanoi, Vietnam; d Guangxi International Zhuang Medicine Hospital Affiliated to Guangxi University of Chinese Medicine, Nanning, Guangxi Province, China.

**Keywords:** acupuncture, children and adolescents, metabolism, obesity/overweight, systematic review and meta-analysis

## Abstract

**Background:**

Although increasing evidence has revealed the efficacy of acupuncture in obesity/overweight, actual improvement in metabolism in children and adolescents is unclear. Therefore, we conducted a meta-analysis to evaluate this correlation.

**Methods:**

A comprehensive search was conducted using multiple databases, including Medline, Cochrane, Embase, Web of Science, Chinese Biomedical Literature Database, China National Knowledge Infrastructure, Chinese Scientific Journal Database, and Wan-fang Data, to identify relevant randomized controlled trials published before February 1, 2023. General information and data for the descriptive and quantitative analyses were extracted.

**Results:**

Fifteen randomized controlled trials of 1288 obese/overweight children and teenagers were included. All the trials were conducted in China and South Korea. Regarding quality assessment, no other significant risk of bias was found. The acupuncture groups were more likely to have improved metabolic indicators of obesity/overweight than the control groups, in terms of body mass index (standardized mean difference [SMD] = −0.45, 95% confidence interval [CI]: −0.69 to −0.21, I2 = 71.4%), body weight (SMD = −0.48, 95% CI: −0.92 to −0.05, I2 = 84.9%), and serum leptin (SMD = −0.34, 95% CI: −0.58 to −0.10, I2 = 91.8%). The subgroup analysis showed that for body mass index, the results were consistent regardless of the intervention duration, body acupuncture or auricular acupuncture combined with other interventions.

**Conclusion:**

Our results suggest that acupuncture is effective in improving metabolic outcomes of obese/overweight children and adolescents. Owing to the limited number of trials included in this study, the results should be interpreted with caution.

## 1. Introduction

Obese/overweight children and adolescents have a nutritional metabolic disorder in which the intake of energy exceeds the requirements for daily activities as well as growth and development, resulting in excessive accumulation or abnormal distribution of body fat, exceeding the standard body mass index (BMI) for the same sex and age.^[1]^ Currently, approximately 20% of children and teenagers worldwide are obese or overweight.^[2]^ The rates of obesity/overweight in children and teens are rising faster than in any other age group, and the World Obesity Federation predicts that these rates will continue to rise significantly, with 208 million boys and 175 million girls becoming obese or overweight by 2035.^[3]^ Childhood obesity/overweight not only increases the risk of mental health problems,^[4]^ but is also strongly associated with cardiovascular disease,^[5]^ increasing the economic burden on families and consuming social resources. Obesity and overweight in children and youth have become one of the most serious global public health challenges.^[6]^

Common treatment options for obesity and overweight include diet control, exercise therapy, drug therapy, and surgical therapy. Diet and exercise therapy are long-term treatments, but because they are difficult to adhere to and need to complement each other, once the diet and exercise are stopped, it is easy to regain weight. Commonly used drugs include metformin and orlistat, which are fast-acting, but they make patients prone to gastrointestinal side effects, insomnia, and other adverse drug reactions.^[7]^ Surgical procedures, such as liposuction and gastric contractions, have obvious curative effects; however, they are expensive, risky, and often cause serious complications.^[8]^ Moreover, children and teenagers are in a vigorous period of growth and development, and it is not advisable to promote excessive dietary restriction, drugs, surgery, and other methods to control the development of obesity/overweight while their height and weight continue to increase. The effective control of obesity/overweight in children and teenagers has become an urgent research task.

Currently, acupuncture is the fastest-developing complementary therapy recognized by the World Health Organization.^[9]^ Acupuncture has attracted considerable attention because of its safety, effectiveness, economy, convenience, and lack of toxic side effects.^[10]^ Several beneficial effects of acupuncture treatment for modulating obesity-related peptide hormones have been reported, including leptin, insulin resistance, lipid metabolism-related hormones,^[11]^ serum immunoglobulin levels, and the hormones that control appetite.^[12]^ Although previous meta-analyses have studied the effectiveness of acupuncture in the treatment of obesity/overweight, evidence on related aspects is lacking and inconsistent, and previous studies also had the following limitations. First, due to the lack of database retrieval and the sample sizes of previous studies on acupuncture for the treatment of obesity/overweight, conclusions regarding the effectiveness of acupuncture may be potentially unreliable.^[13]^ Second, previous studies related to acupuncture for obesity/overweight only considered adults and did not pay much attention to children and teens. However, the problem of obesity/overweight in the latter group has become a public health problem of worldwide concern.^[14]^ Given the lack of studies on the effectiveness of acupuncture in obese/overweight children and teenagers, it is necessary to conduct relevant discussions on this issue. Third, abnormalities in body lipid metabolism are closely related to the development of obesity and overweight. It is necessary to study metabolic abnormalities^[15]^; however, previous meta-analyses of acupuncture for the treatment of obesity/overweight only focused on the outcomes of direct measures of obesity without integrating related metabolic indicators. Thus, evidence-based support for these indicators in children and youth is lacking. The cumulative evidence on acupuncture for obesity/overweight-related metabolic indices in children and teenagers is incomplete and unsystematic, which requires relevant literature to be updated. Therefore, our study aimed to evaluate the effectiveness of acupuncture on metabolic indicators in obese/overweight children and adolescents.

## 2. Methods

Our study was conducted according to the guidelines set out in the Preferred Reporting Items for Systematic Reviews and Meta-Analyses (PRISMA-2020) statement^[16]^ and Cochrane Collaboration Handbook recommendations.^[17]^ We have registered the protocol of this meta-analysis on the International Platform of Registered Systematic Review and Meta analysis Protocols with registration number (INPLASY202370077). Because all analyses were based on previously published studies, no ethical approval or informed consent from patients was required.

Without the restriction of the language of publication, a comprehensive literature search was conducted to identify relevant randomized controlled trials (RCTs) investigating the efficacy of acupuncture in the treatment of obese/overweight children and teenagers using the following electronic databases: Medline (via PubMed), Embase, Cochrane, Web of Science, Chinese National Knowledge Infrastructure, Wan-fang Data, Chinese Scientific Journal Database, and Chinese Biomedical Literature Database, from their inception to February 1, 2023. We employed a combination of the medical subject headings, with free text terms using Boolean logical operators, to create an exhaustive list of search terms. The search terms consist of: “obesity,” “body weight (BW),” “acupuncture,” and “RCT.” In addition, we performed related recursive retrieval as supplementary searches from top journals (China top journals: Acupuncture Research, Chinese Journal of Obesity and Metabolic Diseases, and so on; top international journals: World Journal of Acupuncture-Moxibustion, Obesity, International Journal of Obesity), famous publishers, proceedings of important international conferences, and gray literature (noncommercial bibliography of doctoral and master theses, technical literature [including government reports]) to minimize the loss of missing eligible trials that met the inclusion criteria. References of the included studies, similar meta-analyses, and systematic reviews were also screened to identify suitable studies (shown in Supplementary File 1, http://links.lww.com/MD/J679).

An inspection was necessary for the completeness and accuracy of this study. All initially retrieved records were imported into EndNote X20 (Thomson ISI Research Soft, Philadelphia, Pennsylvania), with which management and confirmation of the above information were performed simultaneously by 2 authors independently. Discrepancies that emerged during this process were resolved by discussion or contacting the corresponding author to confirm the judgments.

### 2.1. Study selection criteria and process

PICOS criteria were used to include studies as follows:

#### 2.1.1. Population.

Participants were obese/overweight children and teenagers within the same age and sex, falling into the category of overweight (BMI ranged between the 85th and 95th percentile) or obesity (BMI ranged exceeding the 95th percentile).^[18,19]^

#### 2.1.2. Interventions.

Acceptable treatments included body acupuncture, auricular acupuncture, and different types of acupuncture combined with other interventions (e.g., exercise, diet, and psychology) as acupuncture groups (EGs).

#### 2.1.3. Comparators.

Subjects undergoing diet, exercise, psychology, and other interventions were included as control groups (CGs), and studies incorporating acupuncture were excluded.

#### 2.1.4. Outcomes.

The primary outcomes were BMI, waist circumference (WC), and BW. Metabolic outcomes included lipid metabolism indicators of obesity/overweight such as serum leptin, triglyceride (TG), total cholesterol (TC), low-density lipoprotein (LDL), and high-density lipoprotein (HDL).

#### 2.1.5. Study selection.

Only published parallel-group RCTs were included, without language restrictions being placed on study selection.

Duplicates were first removed, as defined by our inclusion and exclusion criteria. Two authors independently selected trials by screening the titles and abstracts. Subsequently, a full-text review was conducted to identify potentially eligible trials.

### 2.2. Data extraction and quality assessment

We based data extraction on the Cochrane Consumer and Communications Review Panel guidelines,^[20]^ the 2 authors rigorously screened the data to extract key information for inclusion. According to the pre-specified measures, the following relevant items were collected: major author and publication year, proportion of boys, age, duration of treatment, various outcomes, and so on.

Two independent authors also applied the Cochrane risk of bias (ROB) tool for 7 items to assess the quality of each included RCTs, and each item was evaluated as having a “low,” “unclear,” or “high” ROB. When the generation of random allocation was clearly described and a method for allocation concealment was specified, selection bias was low risk; otherwise, it was high risk. For performance and detection bias, we considered whether the study was blinded to participants, personnel, and outcome assessors; if so, the study was low risk, otherwise it was high risk. For attrition bias, studies with missing data, especially for primary outcomes, were high risk; otherwise, the risk was low. Reporting bias was high risk when studies lacked relevant outcomes or reported deficiencies in available data, such as the characteristics of these outcomes; otherwise, the study was low risk. Other biases were considered high risk if there was concrete evidence that the results were biased by poorly designed studies or by studies that were obviously inconsistent with previous studies; otherwise, they were considered low risk. In the studies, corresponded to items that were not treated in the above 7 items, which were judged as having an “unclear” ROB.^[21]^ Disagreements were resolved through discussion or objective judgment by experienced experts.

### 2.3. Statistical analyses

We conducted the statistical analysis based on the Cochrane Collaboration Handbook recommendations.^[22]^ First, standardized mean difference (SMD) is the difference in the mean with the units removed and effect sizes involving continuous outcomes. The SMD was calculated for each comparison using the group-correlated mean and standard deviation of individual studies, each study with the random-effects model, whose 95% confidence interval (CI) was used to estimate the intervals of the parameters.^[23]^ Second, in terms of statistical heterogeneity, I^2^ statistics with values >25%, 50%, and 75% indicated mild, moderate, and high heterogeneity, respectively, and *P *< .1 was used as the judgment criterion for heterogeneity.^[24]^ Third, the risk of publication bias was suggested if the funnel plot was asymmetrical and the Egger test *P* value was <0.05.^[25]^ Finally, to explore possible sources of heterogeneity, a planned random-effects subgroup analysis based on the variables was conducted. Our model considers a range of domains as follows: intervention duration (>8 vs ≤8 weeks); regional (developed vs backward); publication year (>2015 vs ≤ 2015); total sample size (≥ 70 vs <70); acupuncture combined with diet or not (combined vs not combined); acupuncture combined with exercise or not (combined vs not combined); types of acupuncture (body acupuncture vs auricular acupuncture); acupuncture combined approach (body acupuncture combined with other vs auricular acupuncture combined with other vs auricular acupuncture alone); based on center (outpatient clinic vs school vs community); and status of age (adolescents vs children). All statistical analyses were performed using STATA, version 14.2 (StataCorp, College Station, TX).

## 3. Results

### 3.1. Literature screening results and characteristics of included trials

**Figure 1.**
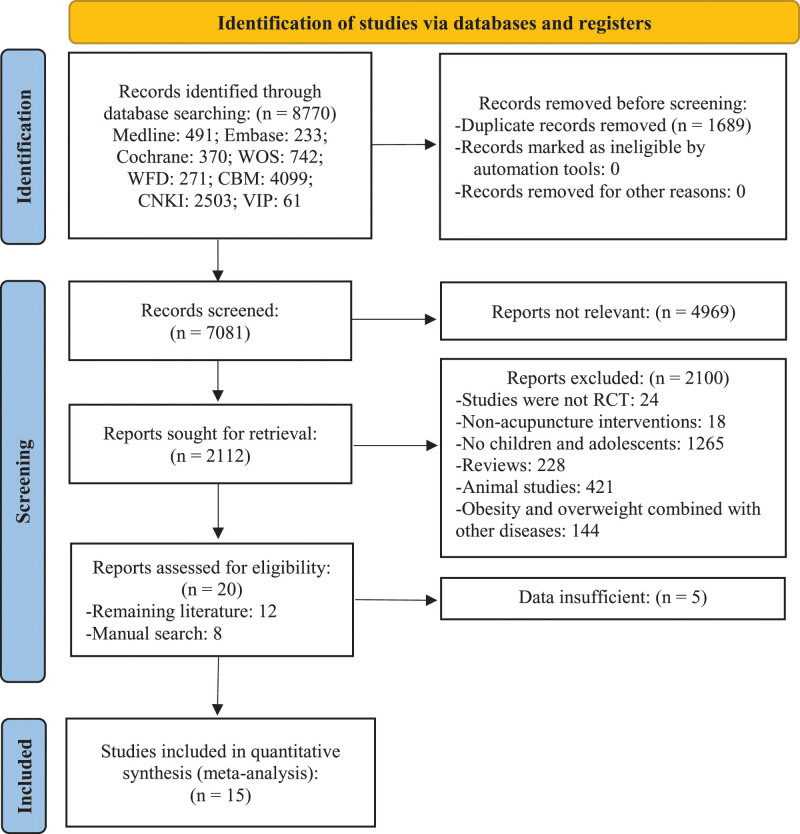
Literature review flowchart. CBM = Chinese biomedical literature database, CNKI = China national knowledge infrastructure database, RCT = randomized controlled trial, VIP = chinese scientific journal database, WFD = Wan-fang database, WOS = web of science.

A total of 8770 records were retrieved from the initial target database, of which 1689 were deleted because of duplication. The manual review identified 8 trials that met the inclusion criteria. In this case, a total of 15 unique trials^[26,27]^ were eventually included in our study (shown in Fig. 1).

All 15 trials were conducted in China and South Korea and published before February 1, 2023. Altogether, 698 and 590 participants were randomized to the EGs and CGs, respectively. The proportion of boys (N = 279) was significantly higher than that of girls (N = 211) (5 studies did not report these data). Different acupuncture approaches were used to intervene in obesity/overweight children and teenagers for 20 days to 3 months (shown in Table 1).

**Table 1:** Characteristics of included studies and participants.

Major author and publication yr	Sample size		Proportion of boys (%)	Age mean ± SD/range		Type of measure for EGs	Type of measure for CGs	Therapeutic duration	Outcomes
	EGs	CGs		EGs	CGs				
Li, 2020^[31]^	27	29	57.1	9.33 ± 1.59	9.24 ± 1.48	Body acupuncture + diet + exercise + massage	Diet + exercise	30 d	①②③
Xiong et al, 2014^[29]^	40	40	60.0	9.37 ± 0.59	9.29 ± 0.46	Body acupuncture + diet + lifestyle + exercise + TCM	Diet + lifestyle + exercise	20 d	①④
Xie et al, 1999^[38]^	35	35	NR	9.00–14.00		Auricular acupuncture + diet + exercise + lifestyle	Diet + exercise + lifestyle	3 mo	③
Li et al, 2006^[37]^	26	30	26.8	16.00 ± 1.38	16.00 ± 1.95	Body acupuncture + diet + exercise + psychology	Diet + exercise + psychology	100 d	①③⑤⑥⑦⑧
Lei, 2006^[30]^	130	30	58.8	6.00–12.00		Body acupuncture + diet	Diet	30 d	①
Yu et al, 2022^[32]^	30	30	58.3	10.01 ± 2.42	9.81 ± 1.40	Body acupuncture + auricular acupuncture + diet + exercise	Diet + exercise	12 wk	①②③
Cha et al, 2019^[27]^	32	26	55.2	16.09 ± 6.00	15.96 ± 5.96	Auricular acupuncture	Placebo	8 wk	①②③④
Cha et al, 2020^[28]^	31	34	NR	10.50 ± 0.99	10.40 ± 0.95	Auricular acupuncture	Placebo	8 wk	①②③
Liu, 2016^[34]^	52	46	53.6	10.34 ± 3.46	10.27 ± 3.54	Auricular acupuncture + meridian points + diet + exercise + psychology	Diet + exercise + psychology	12 wk	①③④
Cao, 2017^[33]^	95	95	59.5	8.01 ± 0.30	7.30 ± 0.30	Auricular acupuncture + diet	Diet	3 mo	①②
Huang et al, 2004^[39]^	30	30	NR	3.00–6.00		Auricular acupuncture + massage	Massage	3 mo	③
Zhu et al, 2000^[40]^	25	20	NR	4.00–6.00		Auricular acupuncture + massage	Massage	12 wk	③
Cao et al, 2021^[26]^	95	95	59.5	8.01 ± 0.30	7.30 ± 0.30	Auricular acupuncture + diet	Diet	3 mo	①
Zhao et al, 2019^[35]^	30	30	NR	7.00–14.00		Auricular acupuncture + diet + exercise + TCM + propaganda and education	Diet + exercise + propaganda and education	3 mo	①
Li et al, 2008^[36]^	20	20	50.0	6.00–17.00		Auricular acupuncture + exercise + lifestyle	Exercise + lifestyle	3 mo	①②④⑤⑥⑦⑧

CGs = control groups, EGs = acupuncture groups, NR = not reported, SD = standard deviation, TCM = traditional Chinese medicine, Outcomes = ① BMI = body mass index, ② WC = waist circumference, ③ BW = body weight, ④ Serum Leptin, ⑤ TG = triglyceride, ⑥ TC = total cholesterol, ⑦ LDL = low-density lipoprotein, ⑧ HDL = high-density lipoprotein.

### 3.2. Quality of included trials

Random assignment was adequately described in all 15 studies; however, none mentioned allocation concealment. The blinding of operators and subjects was mentioned in only 2 studies and the remaining studies did not use blinding or mention it. All 15 trials fully reported the relevant outcomes; therefore, the risk of attrition bias was low (shown in Supplementary Figs. 1 and 2, http://links.lww.com/MD/J680, http://links.lww.com/MD/J681).

### 3.3. Primary outcomes

#### 3.3.1. BMI.

Twelve trials^[26–37]^ investigated the efficacy of EGs and CGs in improving BMI. The results showed that EGs were more effective than CGs in improving BMI, with an SMD value of −0.45 (95% CI: −0.69 to −0.21, I^2^ = 71.4%) (shown in Table 2A). The funnel plot suggested publication bias, but the quantitative Egger test (*P* = .235) showed no publication bias (shown in Supplementary Fig. 3A, http://links.lww.com/MD/J682).

**Table 2:** Primary results based on continuous variable outcomes and subgroup analyses.

A: Primary results based on continuous variable outcomes.						
Primary results based on continuous variable outcomes	No. of studies	No. of participants		Pooled SMDs (95% CI)	Heterogeneity	
		EGs	CGs		I^2^	*P*
BMI	12	608	505	−**0.45** (−**0.69 to** −**0.21**)	71.4%	<.1
WC	6	235	234	0.20 (−0.50 to 0.90)	91.8%	<.1
BW	9	288	280	−**0.56** (−**1.01 to** −**0.10**)	85.7%	<.1
Serum Leptin	4	144	132	−**0.34** (−**0.58 to** −**0.10**)	0.0%	.505
TC	2	46	50	−0.74 (−2.17 to 0.70)	91.1%	<.1
TG	2	46	50	−0.49 (−1.45 to 0.48)	81.0%	<.1
LDL	2	46	50	−**0.45** (−**0.87 to** −**0.03**)	5.7%	.303
HDL	2	46	50	0.06 (−0.34 to 0.46)	0.0%	.842
**B: Subgroup analysis based on the outcome of BMI.**						
**Subgroup analysis based on the outcome of BMI**	**No. of studies**	**No. of participants**		**Pooled SMDs (95% CI)**	**Heterogeneity**	
		**EGs**	**CGs**		**I^2^**	**P**
Intervention duration						
Overall	12	608	505	−**0.45** (−**0.69 to** −**0.21**)	71.4%	<.1
>8 wk	6	322	316	−**0.43** (−**0.77 to** −**0.09**)	75.7%	<.1
≤8 wk	6	286	189	−**0.48** (−**0.83 to** −**0.12**)	68.0%	<.1
Regional						
Overall	12	608	505	−**0.45** (−**0.69 to** −**0.21**)	71.4%	<.1
Develop	8	483	380	−**0.34** (−**0.62 to** −**0.05**)	73.9%	<.1
Backward	4	125	125	−**0.68** (−**0.97 to** −**0.39**)	19.3%	.293
Publication yr						
Overall	12	608	505	−**0.45** (−**0.69 to** −**0.21**)	71.4%	<.1
>2015	8	392	385	−**0.38** (−**0.70 to** −**0.06**)	78.3%	<.1
≤2015	4	216	120	−**0.60** (−**0.84 to** −**0.36**)	0.0%	.899
Total sample size						
Overall	12	608	505	−**0.45** (−**0.69 to** −**0.21**)	71.4%	<.1
≥70	5	412	306	−**0.29** (−**0.54 to** −**0.04**)	60.5%	<.1
<70	7	196	199	−**0.58** (−**0.98 to** −**0.19**)	73.0%	<.1
Acupuncture combined with diet or not						
Overall	12	608	505	−**0.45** (−**0.69 to** −**0.21**)	71.4%	<.1
Combined	9	525	425	−**0.54** (−**0.82 to** −**0.26**)	74.8%	<.1
Not combined	3	83	80	−0.14 (−0.59 to 0.31)	51.7%	.126
Acupuncture combined with exercise or not						
Overall	12	608	505	−**0.45** (−**0.69 to** −**0.21**)	71.4%	<.1
Combined	7	225	225	−**0.73** (−**0.96 to** −**0.51**)	25.7%	.232
Not combined	5	383	280	−0.09 (−0.29 to 0.11)	31.0%	.215
Types of acupuncture						
Overall	12	608	505	−**0.45** (−**0.69 to** −**0.21**)	71.4%	<.1
Body acupuncture	5	253	159	−**0.81** (−**1.10 to −0.52**)	39.6%	.157
Auricular acupuncture	7	355	346	−0.18 (−0.38 to 0.03)	38.6%	.135
Acupuncture combined approach						
Overall	12	608	505	−**0.45** (−**0.69 to** −**0.21**)	71.4%	<.1
Body acupuncture combined with other	5	253	159	−**0.81** (−**1.10 to** −**0.52**)	39.6%	.157
Auricular acupuncture combined with other	5	292	286	−**0.24** (−**0.48 to** −**0.01**)	45.4%	.120
Auricular acupuncture alone	2	63	60	0.04 (−0.35 to 0.43)	16.2%	.275
Based on center						
Overall	12	608	505	−**0.45** (−**0.69 to** −**0.21**)	71.4%	<.1
Outpatient clinic	7	335	235	−**0.70** (−**0.93 to** −**0.48**)	34.0%	.168
School	3	83	80	−0.14 (−0.59 to 0.31)	51.7%	.126
Community	2	190	190	−0.03 (−0.23 to 0.17)	0.0%	1.000
**C: Subgroup analysis based on the outcome of WC.**						
**Subgroup analysis based on the outcome of WC**	**No. of studies**	**No. of participants**		**Pooled SMDs (95% CI)**	**Heterogeneity**	
		**EGs**	**CGs**		**I^2^**	***P***
Intervention duration						
Overall	6	235	234	0.20 (−0.50 to 0.90)	91.8%	<.1
>8 wk	3	145	145	0.52 (−1.01 to 2.06)	96.2%	<.1
≤8 wk	3	90	89	−0.03 (−0.55 to 0.48)	67.2%	<.1
Regional						
Overall	6	235	234	0.20 (−0.50 to 0.90)	91.8%	<.1
Develop	4	188	185	−0.19 (−0.64 to 0.26)	75.8%	<.1
Backward	2	47	49	1.14 (−2.27 to 4.55)	97.6%	<.1
Acupuncture combined with diet or not						
Overall	6	235	234	0.20 (−0.50 to 0.90)	91.8%	<.1
Combined	3	152	154	−**0.56** (−**0.79 to** −**0.33**)	0.0%	.984
Not combined	3	83	80	1.06 (−0.28 to 2.39)	93.2%	<.1
Acupuncture combined with exercise or not						
Overall	6	235	234	0.20 (−0.50 to 0.90)	91.8%	<.1
Combined	3	77	79	0.53 (−1.25 to 2.32)	95.9%	<.1
Not combined	3	158	155	−0.06 (−0.63 to 0.51)	81.6%	<.1
Types of acupuncture						
Overall	6	235	234	0.20 (−0.50 to 0.90)	91.8%	<.1
Body acupuncture	2	57	59	−**0.58** (−**0.96 to** −**0.21**)	0.0%	.982
Auricular acupuncture	4	178	175	0.63 (−0.42 to 1.67)	94.5%	<.1
Acupuncture combined approach						
Overall	6	235	234	0.20 (−0.50 to 0.90)	91.8%	<.1
Body acupuncture combined with other	2	57	59	−**0.58** (−**0.96 to** −**0.21**)	0.0%	.982
Auricular acupuncture combined with other	2	115	115	1.15 (−2.22 to 4.52)	98.0%	<.1
Auricular acupuncture alone	2	63	60	0.23 (−0.13 to 0.58)	0.0%	.886
**D: Subgroup analysis based on the outcome of BW.**						
**Subgroup analysis based on the outcome of BW**	**No. of studies**	**No. of participants**		**Pooled SMDs (95% CI)**	**Heterogeneity**	
		**EGs**	**CGs**		**I^2^**	***P***
Intervention duration						
Overall	9	288	280	−**0.56** (−**1.01 to** −**0.10**)	85.7%	<.1
>8 wk	5	172	161	−**0.90** (−**1.58 to** −**0.22**)	88.2%	<.1
≤8 wk	4	116	119	−0.13 (−0.52 to 0.27)	57.3%	<.1
Regional						
Overall	9	288	280	−**0.56** (−**1.01 to** −**0.10**)	85.7%	<.1
Develop	5	148	140	−0.27 (−0.73 to 0.19)	73.5%	<.1
Backward	4	140	140	−**0.92** (−**1.76 to** −**0.08**)	90.7%	<.1
Publication yr						
Overall	9	288	280	−**0.56** (−**1.01 to** −**0.10**)	85.7%	<.1
>2015	5	172	165	−0.25 (−0.63 to 0.13)	66.8%	<.1
≤2015	4	116	115	−**0.96** (−**1.86 to** −**0.05**)	90.3%	<.1
Total sample size						
Overall	9	288	280	−**0.56** (−**1.01 to** −**0.10**)	85.7%	<.1
≥70	2	87	81	−1.39 (−3.34 to 0.57)	96.5%	<.1
<70	7	201	199	−0.33 (−0.65 to 0.002)	62.6%	<.1
Acupuncture combined with diet or not						
Overall	9	288	280	−**0.56** (−**1.01 to** −**0.10**)	85.7%	<.1
Combined	5	170	170	−**0.89** (−**1.55 to** −**0.24**)	87.6%	<.1
Not combined	4	118	110	−0.13 (−0.61 to 0.34)	69.2%	<.1
Acupuncture combined with exercise or not						
Overall	9	288	280	−**0.56** (−**1.01 to** −**0.10**)	85.7%	<.1
Combined	5	170	170	−**0.89** (−**1.55 to** −**0.24**)	87.6%	<.1
Not combined	4	118	110	−0.13 (−0.61 to 0.34)	69.2%	<.1
Types of acupuncture						
Overall	9	288	280	−**0.56** (−**1.01 to** −**0.10**)	85.7%	<.1
Body acupuncture	3	83	89	−**0.59** (−**0.89 to** −**0.28**)	0.0%	.572
Auricular acupuncture	6	205	191	−0.55 (−1.24 to 0.14)	90.8%	<.1
Acupuncture combined approach						
Overall	9	288	280	−**0.56** (−**1.01 to** −**0.10**)	85.7%	<.1
Body acupuncture combined with other	3	83	89	−**0.59** (−**0.89 to** −**0.28**)	0.0%	.572
Auricular acupuncture combined with other	4	142	131	−**0.92** (−**1.81 to** −**0.04**)	91.2%	<.1
Auricular acupuncture alone	2	63	60	0.20 (−1.15 to 0.56)	0.0%	.513
Based on center						
Overall	9	288	280	−**0.56** (−**1.01 to** −**0.10**)	85.7%	<.1
Outpatient clinic	5	165	165	−**0.57** (−**0.79 to** −**0.35**)	0.0%	.661
School	4	123	115	−0.53 (−1.68 to 0.62)	94.3%	<.1
Status of age						
Overall	9	288	280	−**0.56** (−**1.01 to** −**0.10**)	85.7%	<.1
Adolescents	3	110	102	−0.29 (−0.63 to 0.04)	32.2%	.229
Children	6	178	178	−**0.70** (−**1.40 to** −**0.003**)	89.9%	<.1
**E: Subgroup analysis based on the outcome of Serum Leptin.**						
**Subgroup analysis based on the outcome of Serum Leptin**	**No. of studies**	**No. of participants**		**Pooled SMDs (95% CI)**	**Heterogeneity**	
		**EGs**	**CGs**		**I^2^**	***P***
Intervention duration						
Overall	4	144	132	−**0.34** (−**0.58 to** −**0.10**)	0.0%	.505
>8 wk	2	72	66	−0.31 (−0.65 to 0.02)	0.0%	.411
≤8 wk	2	72	66	−0.36 (−0.79 to 0.07)	37.9%	.205
Regional						
Overall	4	144	132	−**0.34** (−**0.58 to** −**0.10**)	0.0%	.505
Develop	2	72	66	−0.36 (−0.79 to 0.07)	37.9%	.205
Backward	2	72	66	−0.31 (−0.65 to 0.02)	0.0%	.411
Publication yr						
Overall	4	144	132	−**0.34** (−**0.58 to** −**0.10**)	0.0%	.505
>2015	2	84	72	−0.30 (−0.61 to 0.02)	0.0%	.391
≤2015	2	60	60	−0.38 (−0.83 to 0.07)	29.9%	.232
Total sample size						
Overall	4	144	132	−**0.34** (−**0.58 to** −**0.10**)	0.0%	.505
≥70	2	92	86	−**0.47** (−**0.77 to** −**0.18**)	0.0%	.610
<70	2	52	46	−0.11 (−0.51 to 0.29)	0.0%	.956
Acupuncture combined with diet or not						
Overall	4	144	132	−**0.34** (−**0.58 to** −**0.10**)	0.0%	.505
Combined	2	92	86	−**0.47** (−**0.77 to** −**0.18**)	0.0%	.610
Not combined	2	52	46	−0.11 (−0.51 to 0.29)	0.0%	.956
Based on center						
Overall	4	144	132	−**0.34** (−**0.58 to** −**0.10**)	0.0%	.505
Outpatient clinic	2	92	86	−**0.47** (−**0.77 to** −**0.18**)	0.0%	.610
School	2	52	46	−0.11 (−0.51 to 0.29)	0.0%	.956

Bold numbers = statistically significant differences between groups.

BMI = body mass index, BW = body weight, CI = confidence interval, CGs = control groups, EGs = acupuncture groups, HDL = high-density lipoprotein, LDL = low-density lipoprotein, *P* < .1 = heterogeneity exists, SMDs = standard mean differences, TC = total cholesterol, TG = triglyceride, WC = waist circumference.

#### 3.3.2. WC.

Six trials^[27,28,31–33,36]^ investigated the efficacy of EGs and CGs in improving WC, but the results showed no statistical significance (shown in Table 2A). The funnel plot suggested no publication bias, and the quantitative Egger test (*P* = .085) showed the same indication (shown in Supplementary Fig. 3B, http://links.lww.com/MD/J682).

#### 3.3.3. BW.

Nine trials^[27,28,31,32,34,37–40]^ investigated the efficacy of EGs and CGs in weight loss, and the results showed that the efficacy of EGs was better than that of CGs in weight loss, with an SMD value of −0.56 (95% CI: −1.01 to −0.10, I^2^ = 85.7%) (shown in Table 2A). The funnel plot suggested no publication bias, and the quantitative Egger test (*P* = .337) showed the same indication (shown in Supplementary Fig. 3C, http://links.lww.com/MD/J682).

### 3.4. Metabolic outcomes

#### 3.4.1. Serum leptin.

Four trials^[27,29,34,36]^ investigated the efficacy of EGs and CGs in improving serum leptin, and the results showed that EGs were better than CGs in improving serum leptin, with an SMD value of −0.34 (95% CI: −0.58 to −0.10, I^2^ = 91.8%) (shown in Table 2A). The funnel plot suggested no publication bias, and the quantitative Egger test (*P* = .095) showed the same indication (shown in Supplementary Fig. 3D, http://links.lww.com/MD/J682).

#### 3.4.2. Other metabolic indicators.

Two trials^[36,37]^ investigated the efficacy of EGs and CGs in lowering TC, TG, and LDL, and increasing HDL. It was found that EGs were superior to CGs in lowering TC and LDL. The SMD of TC was −0.74 (95% CI: −2.17 to −0.70, I^2^ = 91.8%) and the SMD of LDL was −0.45 (95% CI: −0.87 to −0.03, I^2^ = 5.7%), however, the results of TG and HDL were not statistically different (shown in Table 2A). The corresponding funnel plots all suggested that there was no publication bias (shown in Supplementary Fig. 3E–H, http://links.lww.com/MD/J682).

### 3.5. Subgroup analyses

Based on the primary outcomes, the pre-specified subgroup analyses were divided into 10 categories to explore possible sources of heterogeneity, some of which revealed sources of heterogeneity. In terms of BMI, when regions were considered, the backward region (SMD = −0.68, 95% CI: −0.97, −0.39, I^2^ = 19.3%) had significantly lower heterogeneity than the develop region (SMD = −0.34, 95% CI: −0.62, −0.05, I^2^ = 73.9%). When taking publication year into account, year ≤2015 (SMD = −0.60, 95% CI: −0.84, −0.36, I^2^ = 0.0%) had significantly lower heterogeneity than year >2015 (SMD = −0.38, 95% CI: −0.70, −0.06, I^2^ = 78.3%). In terms of BW, when the types of acupuncture were considered, body acupuncture (SMD = −0.59, 95% CI: −0.89, −0.28, I^2^ = 0.0%) had significantly lower heterogeneity than auricular acupuncture (SMD = −0.92, 95% CI: −1.81, −0.04, I^2^ = 91.2%) (shown in Table 2B–E).

## 4. Discussion

The overall results of this study, which included 15 RCTs and 1288 patients, show that children and adolescents with obesity or overweight assigned to the EGs were more likely to receive more effective treatment than those assigned to the CGs.

For the primary outcomes, the efficacy of EGs on BMI and BW were better than that of CGs: BMI (SMD = −0.45, 95% CI: −0.69 to −0.21), BW (SMD = −0.56, 95% CI: −1.01 to −0.10). Acupuncture can positively regulate the endocrine and digestive systems, which are key to maintaining the efficacy of weight loss and avoiding rebounds.^[41]^ It can improve the fat circulation rate, increase the basal metabolic rate, cause continuous consumption of energy, significantly reduce the thickness of subcutaneous fat or visceral fat, as well as reduce BMI and BW, which is consistent with the results of previous studies with large sample sizes and rigorous methodologies.^[42]^ In the literature included in this study, acupuncture was mostly combined with other interventions, suggesting that the efficacy of acupuncture combined with diet control and appropriate exercise was more obvious.^[43]^ Acupuncture can regulate the hypothalamic center, inhibit the excitation of the feeding center of the hypothalamus, improve the excitability of the satiation center, and reduce hunger, thus reducing food intake.^[44]^ It regulates biological factors such as leptin, growth hormone-releasing peptides, and other biological factors acting on adipocytes, promotes the expression of thermogenic genes, induces mitochondrial biogenesis, increases the browning of white adipose tissue, and increases heat production and energy consumption of the body.^[45]^ Meanwhile, it can enhance oxygen saturation in tissues, boost hormones, and promote body metabolism by stimulating the peripheral and central nervous systems, increasing energy consumption, breaking down excess fat, and reducing the inflammatory response of adipose tissue.^[46]^ Furthermore, by regulating the level of specific bacteria related to obesity, the stability of intestinal flora is improved,^[47]^ and they work together to achieve efficacy in weight loss.

For the related metabolic outcomes, after treatment the levels of TC, LDL and serum leptin were significantly lower in the EGs than in the CGs: serum leptin (SMD = −0.34, 95% CI: −0.58 to −0.10), TC (SMD = −0.74, 95% CI: −2.17 to −0.70), LDL (SMD = −0.45, 95% CI: −0.87 to −0.03). This suggests that acupuncture can effectively regulate energy metabolism through the bidirectional benign regulation of the neuroendocrine system and promote lipid metabolism. The serum leptin level was regulated and there was correct insulin and leptin resistance,^[48]^ thereby lowering blood sugar levels and achieving a balanced state as well as reducing fat synthesis. These results are consistent with the results of previous studies.^[49]^ The therapeutic mechanism of acupuncture is the simultaneous regulation of multiple systems and targets. Acupuncture can improve metabolic indicators by improving the resistance of the “fat-islet endocrine axis,” enhancing the feedback effect of the “fat-insulin axis,” improving insulin sensitivity and normal secretion of insulin in the human body,^[50]^ as well as correcting nerve and endocrine function, so as to improve glucose and lipid metabolism disorders, improve lipoprotein levels, and achieve the purpose of weight loss.^[51]^

The results of subgroup analysis showed that region and publication year may be sources of heterogeneity in BMI. In terms of BW, the different combinations of acupuncture approaches may be a source of heterogeneity. In terms of improving BMI, body acupuncture combined with other therapies was more effective than auricular acupuncture combined with other therapies. However, for BW, the result was reversed, which may be due to individual differences.^[52]^ However, the auricular point sticking method is a noninvasive type of auricular acupuncture that has less stimulation and is less painful. Its efficacy may be weaker than that of other types of acupuncture and it is mostly used for children.^[53]^ Therefore, the efficacy of acupuncture for weight loss may be affected by factors such as region, methods of acupuncture, and individual differences.

This is the first systematic review and meta-analysis focused on establishing the efficacy of acupuncture in the treatment of children and adolescents categorized as overweight and obese. The database search was comprehensive and up to date, more evidence has been added for this age group to make the results more stable and reliable. Our review not only focused on the direct outcomes of obesity/overweight, but also evaluated related metabolic indicators. Overall, our study can be used to improve the evaluation of obesity/overweight to be more objective and comprehensive. The findings of this study may have important implications for the individualized treatment of patients, and we look forward to providing new insights into the treatment of obesity, which may help clinicians, patients, and families make better decisions. Our study also has some limitations. First, the number of RCTs involved in this study was small and the included trials were mostly from China. There were low amounts of original data from other regions. Therefore, the representativeness of the effectiveness of acupuncture to treat obese/overweight children and adolescents was not strong enough. Second, most RCTs had low methodological quality because of inadequate allocation concealment and blinding. Third, differences in treatment duration may have affected the final evaluation of efficacy. Fourth, most studies lacked posttreatment follow-up and thus we could not determine the long-term efficacy of acupuncture, which may add other biases. Furthermore, the auricular acupuncture method is a noninvasive type of acupuncture that has less stimulation and is less painful. Its efficacy may be weaker than that of other types of acupuncture and it is mostly used for children and adolescents. Therefore, relatively speaking, the effect of body acupuncture is better in the treatment of obesity and overweight in children and adolescents. The comparative advantages of different acupuncture intervention modes need to be carried out through network meta-analysis.

## 5. Conclusion

We cautiously concluded that acupuncture can effectively improve direct and metabolic indicators of obesity/overweight in children and adolescents. Moreover, there is still a need for high-quality RCTs to successfully complete more evidence-based studies.

## Acknowledgments

We confirm that this submission is original and has not been submitted for any other form of print or electronic publication. We confirm that each listed author has made a substantial contribution to this work in accordance with the ICMJE guidelines for authorship and is prepared to accept public responsibility. All authors agreed to investigate any misconduct that may be alleged in this work.

## Author contributions

**Data curation:** Tingwei Quan, Qi Su.

**Formal analysis:** Qi Su.

**Funding acquisition:** Hongzhen Tang.

**Investigation:** Jingjun Yang.

**Methodology:** Tingwei Quan, Qi Su.

**Software:** Xin Su.Supervision: Qi Su, Yu Luo, QiuXuan Chen, Hongzhen Tang.

**Writing – original draft:** Tingwei Quan.

**Writing – review & editing:** Tingwei Quan.

## Supplementary Material
