# Supplementary material for: Does acupuncture improve the metabolic outcomes of obese/overweight children and adolescents?: A systematic review and meta-analysis
Source: Medicine (Baltimore). 2023 Oct 6;102(40):e34943. doi: 10.1097/MD.0000000000034943 (PMC10552954; doi:10.1097/MD.0000000000034943)
Supplement: Supplementary file 1 [file medi-102-e34943-s001.docx]

**Supplementary File.1 Search strategy**

**Medline strategy**

1. "Obesity"[Mesh] 253,528
2. (((((((((((((((((((((((((((((((((((((((((((((((((((((((((((((((((((((((((((((((((((Appetite Depressants) OR (Depressants, Appetite)) OR (Anorectic Agents)) OR (Agents, Anorectic)) OR (Anorectic Agent)) OR (Agent, Anorectic)) OR (Anorexic Drug)) OR (Drug, Anorexic)) OR (Anorexigenic Drug)) OR (Drug, Anorexigenic)) OR (Appetite Suppressant)) OR (Suppressant, Appetite)) OR (Anorectics)) OR (Appetite Suppressants)) OR (Suppressants, Appetite)) OR (Appetite-Depressing Drug)) OR (Appetite Depressing Drug)) OR (Drug, Appetite-Depressing)) OR (Appetite-Suppressant Drug)) OR (Appetite Suppressant Drug)) OR (Drug, Appetite-Suppressant)) OR (Anorexigenic Drugs)) OR (Drugs, Anorexigenic)) OR (Appetite-Depressing Drugs)) OR (Appetite Depressing Drugs)) OR (Drugs, Appetite-Depressing)) OR (Anorexic Drugs)) OR (Drugs, Anorexic)) OR (Appetite-Suppressant Drugs)) OR (Appetite Suppressant Drugs)) OR (Drugs, Appetite-Suppressant)) OR (Anorectic)) OR (Appetite Depressant)) OR (Depressant, Appetite)) OR (Body Weight)) OR (Body Weights)) OR (Weight, Body)) OR (Weights, Body)) OR (Diet, Reducing)) OR (Diets, Reducing)) OR (Reducing Diet)) OR (Reducing Diets)) OR (Weight Reduction Diet)) OR (Diet, Weight Reduction)) OR (Diets, Weight Reduction)) OR (Weight Reduction Diets)) OR (Weight Loss Diet)) OR (Diet, Weight Loss)) OR (Diets, Weight Loss)) OR (Weight Loss Diets)) OR (Skinfold Thickness)) OR (Skinfold Thicknesses)) OR (Thickness, Skinfold)) OR (Thicknesses, Skinfold)) OR (Anti-Obesity Agents)) OR (Agents, Anti-Obesity)) OR (Anti Obesity Agents)) OR (Anti-Obesity Drugs)) OR (Anti Obesity Drugs)) OR (Drugs, Anti-Obesity)) OR (Anti-Obesity Agent)) OR (Agent, Anti-Obesity)) OR (Anti Obesity Agent)) OR (Antiobesity Agent)) OR (Agent, Antiobesity)) OR (Antiobesity Drugs)) OR (Drugs, Antiobesity)) OR (Anti-Obesity Drug)) OR (Anti Obesity Drug)) OR (Drug, Anti-Obesity)) OR (Antiobesity Agents)) OR (Agents, Antiobesity)) OR (Antiobesity Drug)) OR (Drug, Antiobesity)) OR (Weight-Loss Agents)) OR (Agents, Weight-Loss)) OR (Weight Loss Agents)) OR (Weight-Loss Drugs)) OR (Drugs, Weight-Loss)) OR (Weight Loss Drugs)) OR (Weight-Loss Drug)) OR (Drug, Weight-Loss)) OR (Weight Loss Drug)) OR (Bariatrics) 820,333
3. ("Obesity"[Mesh]) OR ((((((((((((((((((((((((((((((((((((((((((((((((((((((((((((((((((((((((((((((((((((Appetite Depressants) OR (Depressants, Appetite)) OR (Anorectic Agents)) OR (Agents, Anorectic)) OR (Anorectic Agent)) OR (Agent, Anorectic)) OR (Anorexic Drug)) OR (Drug, Anorexic)) OR (Anorexigenic Drug)) OR (Drug, Anorexigenic)) OR (Appetite Suppressant)) OR (Suppressant, Appetite)) OR (Anorectics)) OR (Appetite Suppressants)) OR (Suppressants, Appetite)) OR (Appetite-Depressing Drug)) OR (Appetite Depressing Drug)) OR (Drug, Appetite-Depressing)) OR (Appetite-Suppressant Drug)) OR (Appetite Suppressant Drug)) OR (Drug, Appetite-Suppressant)) OR (Anorexigenic Drugs)) OR (Drugs, Anorexigenic)) OR (Appetite-Depressing Drugs)) OR (Appetite Depressing Drugs)) OR (Drugs, Appetite-Depressing)) OR (Anorexic Drugs)) OR (Drugs, Anorexic)) OR (Appetite-Suppressant Drugs)) OR (Appetite Suppressant Drugs)) OR (Drugs, Appetite-Suppressant)) OR (Anorectic)) OR (Appetite Depressant)) OR (Depressant, Appetite)) OR (Body Weight)) OR (Body Weights)) OR (Weight, Body)) OR (Weights, Body)) OR (Diet, Reducing)) OR (Diets, Reducing)) OR (Reducing Diet)) OR (Reducing Diets)) OR (Weight Reduction Diet)) OR (Diet, Weight Reduction)) OR (Diets, Weight Reduction)) OR (Weight Reduction Diets)) OR (Weight Loss Diet)) OR (Diet, Weight Loss)) OR (Diets, Weight Loss)) OR (Weight Loss Diets)) OR (Skinfold Thickness)) OR (Skinfold Thicknesses)) OR (Thickness, Skinfold)) OR (Thicknesses, Skinfold)) OR (Anti-Obesity Agents)) OR (Agents, Anti-Obesity)) OR (Anti Obesity Agents)) OR (Anti-Obesity Drugs)) OR (Anti Obesity Drugs)) OR (Drugs, Anti-Obesity)) OR (Anti-Obesity Agent)) OR (Agent, Anti-Obesity)) OR (Anti Obesity Agent)) OR (Antiobesity Agent)) OR (Agent, Antiobesity)) OR (Antiobesity Drugs)) OR (Drugs, Antiobesity)) OR (Anti-Obesity Drug)) OR (Anti Obesity Drug)) OR (Drug, Anti-Obesity)) OR (Antiobesity Agents)) OR (Agents, Antiobesity)) OR (Antiobesity Drug)) OR (Drug, Antiobesity)) OR (Weight-Loss Agents)) OR (Agents, Weight-Loss)) OR (Weight Loss Agents)) OR (Weight-Loss Drugs)) OR (Drugs, Weight-Loss)) OR (Weight Loss Drugs)) OR (Weight-Loss Drug)) OR (Drug, Weight-Loss)) OR (Weight Loss Drug)) OR (Bariatrics)) 823,345
4. "Acupuncture"[Mesh] 1,999
5. (((((Pharmacopuncture) OR (Acupuncture Therapy)) OR (Acupuncture Treatment)) OR (Acupuncture Treatments)) OR (Treatment, Acupuncture)) OR (Therapy, Acupuncture)

40,719

1. ("Acupuncture"[Mesh]) OR ((((((Pharmacopuncture) OR (Acupuncture Therapy)) OR (Acupuncture Treatment)) OR (Acupuncture Treatments)) OR (Treatment, Acupuncture)) OR (Therapy, Acupuncture)) 40,719
2. (randomized controlled trial [pt] OR controlled clinical trial [pt] OR randomized [tiab] OR placebo [tiab] OR drug therapy [sh] OR randomly [tiab] OR trial [tiab] OR groups [tiab]) NOT (animals [mh] NOT humans [mh]) 4,917,826
3. ((("Obesity"[Mesh]) OR ((((((((((((((((((((((((((((((((((((((((((((((((((((((((((((((((((((((((((((((((((((Appetite Depressants) OR (Depressants, Appetite)) OR (Anorectic Agents)) OR (Agents, Anorectic)) OR (Anorectic Agent)) OR (Agent, Anorectic)) OR (Anorexic Drug)) OR (Drug, Anorexic)) OR (Anorexigenic Drug)) OR (Drug, Anorexigenic)) OR (Appetite Suppressant)) OR (Suppressant, Appetite)) OR (Anorectics)) OR (Appetite Suppressants)) OR (Suppressants, Appetite)) OR (Appetite-Depressing Drug)) OR (Appetite Depressing Drug)) OR (Drug, Appetite-Depressing)) OR (Appetite-Suppressant Drug)) OR (Appetite Suppressant Drug)) OR (Drug, Appetite-Suppressant)) OR (Anorexigenic Drugs)) OR (Drugs, Anorexigenic)) OR (Appetite-Depressing Drugs)) OR (Appetite Depressing Drugs)) OR (Drugs, Appetite-Depressing)) OR (Anorexic Drugs)) OR (Drugs, Anorexic)) OR (Appetite-Suppressant Drugs)) OR (Appetite Suppressant Drugs)) OR (Drugs, Appetite-Suppressant)) OR (Anorectic)) OR (Appetite Depressant)) OR (Depressant, Appetite)) OR (Body Weight)) OR (Body Weights)) OR (Weight, Body)) OR (Weights, Body)) OR (Diet, Reducing)) OR (Diets, Reducing)) OR (Reducing Diet)) OR (Reducing Diets)) OR (Weight Reduction Diet)) OR (Diet, Weight Reduction)) OR (Diets, Weight Reduction)) OR (Weight Reduction Diets)) OR (Weight Loss Diet)) OR (Diet, Weight Loss)) OR (Diets, Weight Loss)) OR (Weight Loss Diets)) OR (Skinfold Thickness)) OR (Skinfold Thicknesses)) OR (Thickness, Skinfold)) OR (Thicknesses, Skinfold)) OR (Anti-Obesity Agents)) OR (Agents, Anti-Obesity)) OR (Anti Obesity Agents)) OR (Anti-Obesity Drugs)) OR (Anti Obesity Drugs)) OR (Drugs, Anti-Obesity)) OR (Anti-Obesity Agent)) OR (Agent, Anti-Obesity)) OR (Anti Obesity Agent)) OR (Antiobesity Agent)) OR (Agent, Antiobesity)) OR (Antiobesity Drugs)) OR (Drugs, Antiobesity)) OR (Anti-Obesity Drug)) OR (Anti Obesity Drug)) OR (Drug, Anti-Obesity)) OR (Antiobesity Agents)) OR (Agents, Antiobesity)) OR (Antiobesity Drug)) OR (Drug, Antiobesity)) OR (Weight-Loss Agents)) OR (Agents, Weight-Loss)) OR (Weight Loss Agents)) OR (Weight-Loss Drugs)) OR (Drugs, Weight-Loss)) OR (Weight Loss Drugs)) OR (Weight-Loss Drug)) OR (Drug, Weight-Loss)) OR (Weight Loss Drug)) OR (Bariatrics))) AND (("Acupuncture"[Mesh]) OR ((((((Pharmacopuncture) OR (Acupuncture Therapy)) OR (Acupuncture Treatment)) OR (Acupuncture Treatments)) OR (Treatment, Acupuncture)) OR (Therapy, Acupuncture)))) AND ((randomized controlled trial [pt] OR controlled clinical trial [pt] OR randomized [tiab] OR placebo [tiab] OR drug therapy [sh] OR randomly [tiab] OR trial [tiab] OR groups [tiab]) NOT (animals [mh] NOT humans [mh])) 491

**Embase strategy**

1. 'obesity'/exp 636,925
2. 'appetite depressants':ti,ab,kw OR 'depressants,appetite':ti,ab,kw OR 'anorectic agents':ti,ab,kw OR 'agents, anorectic':ti,ab,kw OR 'anorectic agent':ti,ab,kw OR 'agent, anorectic':ti,ab,kw OR 'anorexic drug':ti,ab,kw OR 'drug, anorexic':ti,ab,kw OR 'anorexigenic drug':ti,ab,kw OR 'drug, anorexigenic':ti,ab,kw OR 'appetite suppressant':ti,ab,kw OR 'suppressant, appetite':ti,ab,kw OR 'anorectics':ti,ab,kw OR 'appetite suppressants':ti,ab,kw OR 'suppressants, appetite':ti,ab,kw OR 'appetite-depressing drug':ti,ab,kw OR 'appetite depressing drug':ti,ab,kw OR 'drug, appetite-depressing':ti,ab,kw OR 'appetite-suppressant drug':ti,ab,kw OR 'appetite suppressant drug':ti,ab,kw OR 'drug, appetite-suppressant':ti,ab,kw OR 'anorexigenic drugs':ti,ab,kw OR 'drugs, anorexigenic':ti,ab,kw OR 'appetite-depressing drugs':ti,ab,kw OR 'appetite depressing drugs':ti,ab,kw OR 'drugs, appetite-depressing':ti,ab,kw OR 'anorexic drugs':ti,ab,kw OR 'drugs, anorexic':ti,ab,kw OR 'appetite-suppressant drugs':ti,ab,kw OR 'appetite suppressant drugs':ti,ab,kw OR 'drugs, appetite-suppressant':ti,ab,kw OR 'anorectic':ti,ab,kw OR 'appetite depressant':ti,ab,kw OR 'depressant, appetite':ti,ab,kw OR 'body weight':ti,ab,kw OR 'body weights':ti,ab,kw OR 'weight, body':ti,ab,kw OR 'weights, body':ti,ab,kw OR 'diet, reducing':ti,ab,kw OR 'diets, reducing':ti,ab,kw OR 'reducing diet':ti,ab,kw OR 'reducing diets':ti,ab,kw OR 'weight reduction diet':ti,ab,kw OR 'diet, weight reduction':ti,ab,kw OR 'diets, weight reduction':ti,ab,kw OR 'weight reduction diets':ti,ab,kw OR 'weight loss diet':ti,ab,kw OR 'diet, weight loss':ti,ab,kw OR 'diets, weight loss':ti,ab,kw OR 'weight loss diets':ti,ab,kw OR 'skinfold thickness':ti,ab,kw OR 'skinfold thicknesses':ti,ab,kw OR 'thickness, skinfold':ti,ab,kw OR 'thicknesses, skinfold':ti,ab,kw OR 'anti-obesity agents':ti,ab,kw OR 'agents, anti-obesity':ti,ab,kw OR 'anti obesity agents':ti,ab,kw OR 'anti-obesity drugs':ti,ab,kw OR 'anti obesity drugs':ti,ab,kw OR 'drugs,anti-obesity':ti,ab,kw OR 'anti-obesity agent':ti,ab,kw OR 'agent, anti-obesity':ti,ab,kw OR 'anti obesity agent':ti,ab,kw OR 'antiobesity agent':ti,ab,kw OR 'agent, antiobesity':ti,ab,kw OR 'antiobesity drugs':ti,ab,kw OR 'drugs, antiobesity':ti,ab,kw OR 'anti-obesity drug':ti,ab,kw OR 'anti obesity drug':ti,ab,kw OR 'drug, anti-obesity':ti,ab,kw OR 'antiobesity agents':ti,ab,kw OR 'agents, antiobesity':ti,ab,kw OR 'antiobesity drug':ti,ab,kw OR 'drug, antiobesity':ti,ab,kw OR 'weight-loss agents':ti,ab,kw OR 'agents, weight-loss':ti,ab,kw OR 'weight loss agents':ti,ab,kw OR 'weight-loss drugs':ti,ab,kw OR 'drugs, weight-loss':ti,ab,kw OR 'weight loss drugs':ti,ab,kw OR 'weight-loss drug':ti,ab,kw OR 'drug, weight-loss':ti,ab,kw OR 'weight loss drug':ti,ab,kw OR 'bariatrics':ti,ab,kw 346,342
3. 'acupuncture'/exp 55,614
4. #1 OR #2 920,022
5. 'pharmacopuncture':ti,ab,kw OR 'acupuncture therapy':ti,ab,kw OR 'acupuncture treatment':ti,ab,kw OR 'acupuncture treatments':ti,ab,kw OR 'treatment, acupuncture':ti,ab,kw OR 'therapy, acupuncture':ti,ab,kw 7,486
6. #3 OR #5 55,993
7. ('crossover procedure':de OR 'double-blind procedure':de OR 'randomized controlled trial':de) AND or  AND 'single-blind procedure':de OR (random*:de,ab,ti AND or :de,ab,ti AND factorial*:de,ab,ti) OR crossover*:de,ab,ti OR ((cross NEXT/1 over*):de,ab,ti) OR placebo*:de,ab,ti OR ((doubl* NEAR/1 blind*):de,ab,ti) OR ((singl* NEAR/1 blind*):de,ab,ti) OR assign*:de,ab,ti OR allocat*:de,ab,ti OR volunteer*:de,ab,ti 1,589,948
8. #4 AND #6 AND #7 233

**Cochrane search strategy**

1. MeSH descriptor: [Obesity] explode all trees 16,226
2. (Appetite Depressants):ti,ab,kw OR (Depressants, Appetite):ti,ab,kw OR (Anorectic Agents):ti,ab,kw OR (Agents, Anorectic):ti,ab,kw OR (Anorectic Agent):ti,ab,kw OR (Agent, Anorectic):ti,ab,kw OR (Anorexic Drug):ti,ab,kw OR (Drug, Anorexic):ti,ab,kw OR (Anorexigenic Drug):ti,ab,kw OR (Drug, Anorexigenic):ti,ab,kw OR (Appetite Suppressant):ti,ab,kw OR (Suppressant, Appetite):ti,ab,kw OR (Anorectics):ti,ab,kw OR (Appetite Suppressants):ti,ab,kw OR (Suppressants, Appetite):ti,ab,kw OR (Appetite-Depressing Drug):ti,ab,kw OR (Appetite Depressing Drug):ti,ab,kw OR (Drug, Appetite-Depressing):ti,ab,kw OR (Appetite-Suppressant Drug):ti,ab,kw OR (Appetite Suppressant Drug):ti,ab,kw 624
3. (Drug, Appetite-Suppressant):ti,ab,kw OR (Anorexigenic Drugs):ti,ab,kw OR (Drugs, Anorexigenic):ti,ab,kw OR (Appetite-Depressing Drugs):ti,ab,kw OR (Appetite Depressing Drugs):ti,ab,kw OR (Drugs, Appetite-Depressing):ti,ab,kw OR (Anorexic Drugs):ti,ab,kw OR (Drugs, Anorexic):ti,ab,kw OR (Appetite-Suppressant Drugs):ti,ab,kw OR (Appetite Suppressant Drugs):ti,ab,kw OR (Drugs, Appetite-Suppressant):ti,ab,kw OR (Anorectic):ti,ab,kw OR (appetite depressant):ti,ab,kw OR (Depressant, Appetite):ti,ab,kw OR (Body Weight):ti,ab,kw OR (Body Weights):ti,ab,kw OR (Weight, Body):ti,ab,kw OR (Weights, Body):ti,ab,kw OR (Diet, Reducing):ti,ab,kw OR (Diets, Reducing):ti,ab,kw OR (Reducing Diet):ti,ab,kw OR (Reducing Diets):ti,ab,kw OR (Weight Reduction Diet):ti,ab,kw OR (Diet, Weight Reduction):ti,ab,kw OR (Diets, Weight Reduction):ti,ab,kw 81,253
4. (Weight Reduction Diets):ti,ab,kw OR (Weight Loss Diet):ti,ab,kw OR (Diet, Weight Loss):ti,ab,kw OR (Diets, Weight Loss):ti,ab,kw OR (Weight Loss Diets):ti,ab,kw OR (Skinfold Thickness):ti,ab,kw OR (Skinfold Thicknesses):ti,ab,kw OR (Thickness, Skinfold):ti,ab,kw OR (Thicknesses, Skinfold):ti,ab,kw OR (Anti-Obesity Agents):ti,ab,kw OR (Agents, Anti-Obesity):ti,ab,kw OR (Anti Obesity Agents):ti,ab,kw OR (Anti-Obesity Drugs):ti,ab,kw OR (Anti Obesity Drugs):ti,ab,kw OR (Drugs, Anti-Obesity):ti,ab,kw OR (Anti-Obesity Agent):ti,ab,kw OR (Agent, Anti-Obesity):ti,ab,kw OR (Anti Obesity Agent):ti,ab,kw OR (Antiobesity Agent):ti,ab,kw OR (Agent, Antiobesity):ti,ab,kw OR (Antiobesity Drugs):ti,ab,kw OR (Drugs, Antiobesity):ti,ab,kw OR (Anti-Obesity Drug):ti,ab,kw OR (Anti Obesity Drug):ti,ab,kw OR (Drug, Anti-Obesity):ti,ab,kw OR (Antiobesity Agents):ti,ab,kw OR (Agents, Antiobesity):ti,ab,kw OR (Antiobesity Drug):ti,ab,kw OR (Drug, Antiobesity):ti,ab,kw OR (Weight-Loss Agents):ti,ab,kw OR (Agents, Weight-Loss):ti,ab,kw OR (Weight Loss Agents):ti,ab,kw OR (Weight-Loss Drugs):ti,ab,kw OR (Drugs, Weight-Loss):ti,ab,kw OR (Weight Loss Drugs):ti,ab,kw 16,113
5. #1 OR #2 OR #3 OR #4 92,108
6. MeSH descriptor: [Acupuncture] explode all trees 167
7. (Pharmacopuncture):ti,ab,kw OR (Acupuncture Therapy):ti,ab,kw OR (Acupuncture Treatment):ti,ab,kw OR (Acupuncture Treatments):ti,ab,kw OR (Treatment, Acupuncture):ti,ab,kw OR (Therapy,Acupuncture):ti,ab,kw 14,029
8. #6 OR #7 14,047
9. #5 AND #8 370

**Web of Science search strategy**

1. "TS=(Obesity OR Appetite Depressants OR Depressants, Appetite OR Anorectic Agents OR Agents, Anorectic OR Anorectic Agent OR Agent, Anorectic OR Anorexic Drug OR Drug, Anorexic OR Anorexigenic Drug OR Drug, Anorexigenic OR Appetite Suppressant OR Suppressant, Appetite OR Anorectics OR Appetite Suppressants OR Suppressants, Appetite OR Appetite-Depressing Drug OR Appetite Depressing Drug OR Drug, Appetite-Depressing OR Appetite-Suppressant Drug OR Appetite Suppressant Drug OR Drug, Appetite-Suppressant OR Anorexigenic Drugs OR Drugs, Anorexigenic OR Appetite-Depressing Drugs OR Appetite Depressing Drugs OR Drugs, Appetite-Depressing OR Anorexic Drugs OR Drugs, Anorexic OR Appetite-Suppressant Drugs OR Appetite Suppressant Drugs OR Drugs, Appetite-Suppressant OR Anorectic OR Appetite Depressant OR Depressant, Appetite OR Body Weight OR Body Weights OR Weight, Body OR Weights, Body OR Diet, Reducing OR Diets, Reducing OR Reducing Diet OR Reducing Diets OR Weight Reduction Diet OR Diet, Weight Reduction OR Diets, Weight Reduction OR Weight Reduction Diets OR Weight Loss Diet OR Diet, Weight Loss OR Diets, Weight Loss OR Weight Loss Diets OR Skinfold Thickness OR Skinfold Thicknesses OR Thickness, Skinfold OR Thicknesses, Skinfold OR Anti-Obesity Agents OR Agents, Anti-Obesity OR Anti Obesity Agents OR Anti-Obesity Drugs OR Anti Obesity Drugs OR Drugs, Anti-Obesity OR Anti-Obesity Agent OR Agent, Anti-Obesity OR Anti Obesity Agent OR Antiobesity Agent OR Agent, Antiobesity OR Antiobesity Drugs OR Drugs, Antiobesity OR Anti-Obesity Drug OR Anti Obesity Drug OR Drug, Anti-Obesity OR Antiobesity Agents OR Agents, Antiobesity OR Antiobesity Drug OR Drug, Antiobesity OR Weight-Loss Agents OR Agents, Weight-Loss OR Weight Loss Agents OR Weight-Loss Drugs OR Drugs, Weight-Loss OR Weight Loss Drugs OR Weight-Loss Drug OR Drug, Weight-Loss OR Weight Loss Drug OR Bariatrics)" 1,441,894
2. "TS=(Acupuncture OR Pharmacopuncture OR Acupuncture Therapy OR Acupuncture Treatment OR Acupuncture Treatments OR Treatment, Acupuncture OR Therapy, Acupuncture)" 40,102
3. "TS=clinical trial* OR TS=research design OR TS=comparative stud* OR TS=evaluation stud* OR TS=controlled trial* OR TS=follow-up stud* OR TS=prospective stud* OR TS=random* OR TS=placebo* OR TS=(single blind*) OR TS=(double blind*)" 10,468,580
4. AND #2 AND #1 742

**CBM search strategy**

1. "肥胖症"[不加权:扩展] 42,319
2. 肥胖 OR 超重 OR 减肥 OR 体重 OR 减重 OR 减脂 OR 降脂 239,815
3. "针灸疗法"[不加权:扩展] 159,871
4. 针刺 OR 针灸 OR 刺灸 OR 温针 OR 电针 OR 针 OR 灸 OR 耳穴 OR 耳针 OR 埋线 810,552
5. (#3) OR (#2) 239,906
6. (#5) OR (#4) 810,552
7. "随机对照试验"[不加权:扩展] 199,665
8. 随机 1,819,387
9. (#9) OR (#8) 1,819,582

#10. (#10) AND (#7) AND (#6) 4,099

**VIP search strategy**

1. 题名或关键词：肥胖 OR 超重 OR 减肥 OR 体重 OR 减重 OR 减脂 OR 降脂

**AND**

题名或关键词：针刺 OR 针灸 OR 刺灸 OR 温针 OR 电针 OR 针 OR 灸 OR 耳穴 OR 耳针OR 埋线

**AND**

任意字段： 随机对照研究 OR 随机对照实验 OR 随机对照试验 OR 随机对照 OR 随机 OR RCT 61

**CNKI search strategy**

1. （主题 + 中英文扩展）：肥胖 + 超重 + 减肥 + 体重 + 减重 + 减脂 + 降脂

**AND**

（主题 + 中英文扩展）：针刺 + 针灸 + 刺灸 + 温针 + 电针 + 针 + 灸 + 耳针 + 埋线

**AND**

（篇关摘 + 中英文扩展）：随机对照试验 + 随机对照实验 + 随机对照研究 + 随机对照 + 随机 + RCT 2,504

**Wan-fang Data search strategy**

1. （主题 + 中英文扩展）：肥胖 or 超重 or 减肥 or 体重 or 减重 or 减脂 or 降脂

**AND**

（主题 + 中英文扩展）：针刺 or 针灸 or 刺灸 or 温针 or 电针 or针 or 灸 or 耳穴 or 耳针 or 埋线

**AND**

（题名或关键词）：随机对照研究 or 随机对照实验 or 随机对照 or 随机对照 or 随机 or RCT 271
