# Supplementary material for: Does acupuncture improve the metabolic outcomes of obese/overweight children and adolescents?: A systematic review and meta-analysis
Source: Medicine (Baltimore). 2023 Oct 6;102(40):e34943. doi: 10.1097/MD.0000000000034943 (PMC10552954; doi:10.1097/MD.0000000000034943)
Supplement: Supplementary file 2 [file medi-102-e34943-s002.docx]

| Random allocation  (selection bias) | L | L | L | L | L | L | L | L | L | L | L | L | L | L | L |
| --- | --- | --- | --- | --- | --- | --- | --- | --- | --- | --- | --- | --- | --- | --- | --- |
| Allocation concealment  (selection bias) | U | U | U | U | U | U | U | U | U | U | U | U | U | U | U |
| Blinding of participants and personnel (performance bias) | H | H | H | U | H | U | H | H | U | H | L | H | H | U | L |
| Blinding of outcome assessors (detection bias) | L | H | H | L | H | U | H | H | U | H | L | H | H | U | L |
| Attrition bias | L | L | L | L | L | L | L | L | L | L | L | L | L | L | L |
| Reporting bias | U | U | U | U | U | U | U | U | U | U | U | U | U | U | U |
| Other bias | L | L | L | L | L | L | L | L | L | L | L | L | L | L | L |
| 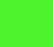 **High risk**  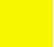 **Unclear risk**  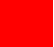 **High risk** | Li, 2020 | Xiong et al, 2014 | Xie et al, 1999 | Li et al, 2006 | Lei, 2006 | Yu et al, 2022 | Cha et al, 2019 | Cha et al, 2020 | Liu, 2016 | Cao, 2017 | Huang et al, 2004 | Zhu et al, 2000 | Cao et al, 2021 | Zhao et al, 2019 | Li et al, 2008 |

**Supplementary Figure.1 Risk of bias graph**
