# Supplementary material for: Does acupuncture improve the metabolic outcomes of obese/overweight children and adolescents?: A systematic review and meta-analysis
Source: Medicine (Baltimore). 2023 Oct 6;102(40):e34943. doi: 10.1097/MD.0000000000034943 (PMC10552954; doi:10.1097/MD.0000000000034943)
Supplement: Supplementary file 3 [file medi-102-e34943-s003.docx]

**Supplementary Figure.2 Risk of bias summary**
