# Supplementary material for: Does acupuncture improve the metabolic outcomes of obese/overweight children and adolescents?: A systematic review and meta-analysis
Source: Medicine (Baltimore). 2023 Oct 6;102(40):e34943. doi: 10.1097/MD.0000000000034943 (PMC10552954; doi:10.1097/MD.0000000000034943)
Supplement: Supplementary file 4 [file medi-102-e34943-s004.docx]

**Supplementary Figure.3 Funnel plots of outcomes**

**A: Funnel plot of BMI (body mass index)**


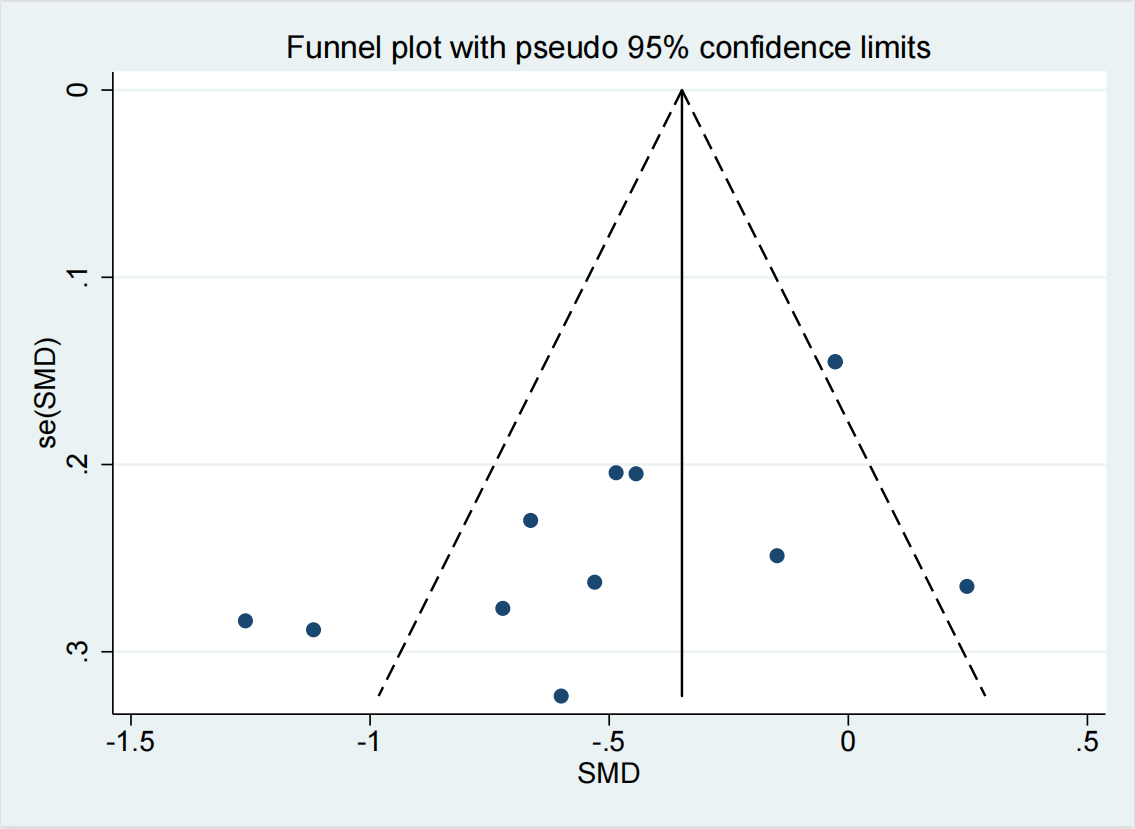


The abscissa is the effect size standard mean difference (SMD), the ordinate is the standard error of the effect size, the dots represent the studies included; the funnel plot is asymmetrical, four dots fell outside the 95% confidence interval, Egger’s test *P* = 0.235.

**B: Funnel plot of WC (waist circumference)**


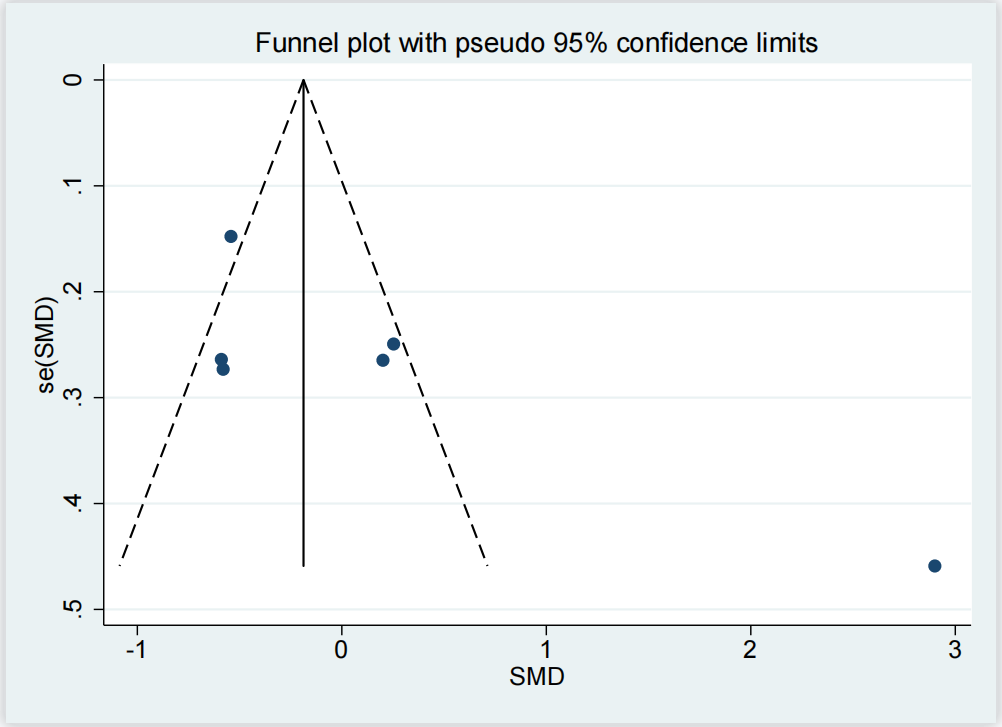


The abscissa is the effect size standard mean difference (SMD), the ordinate is the standard error of the effect size, the dots represent the studies included; funnel plot is symmetry, two dots fell outside the 95% confidence interval, Egger’s test *P* = 0.085.

**C: Funnel plot of BW (body weight)**


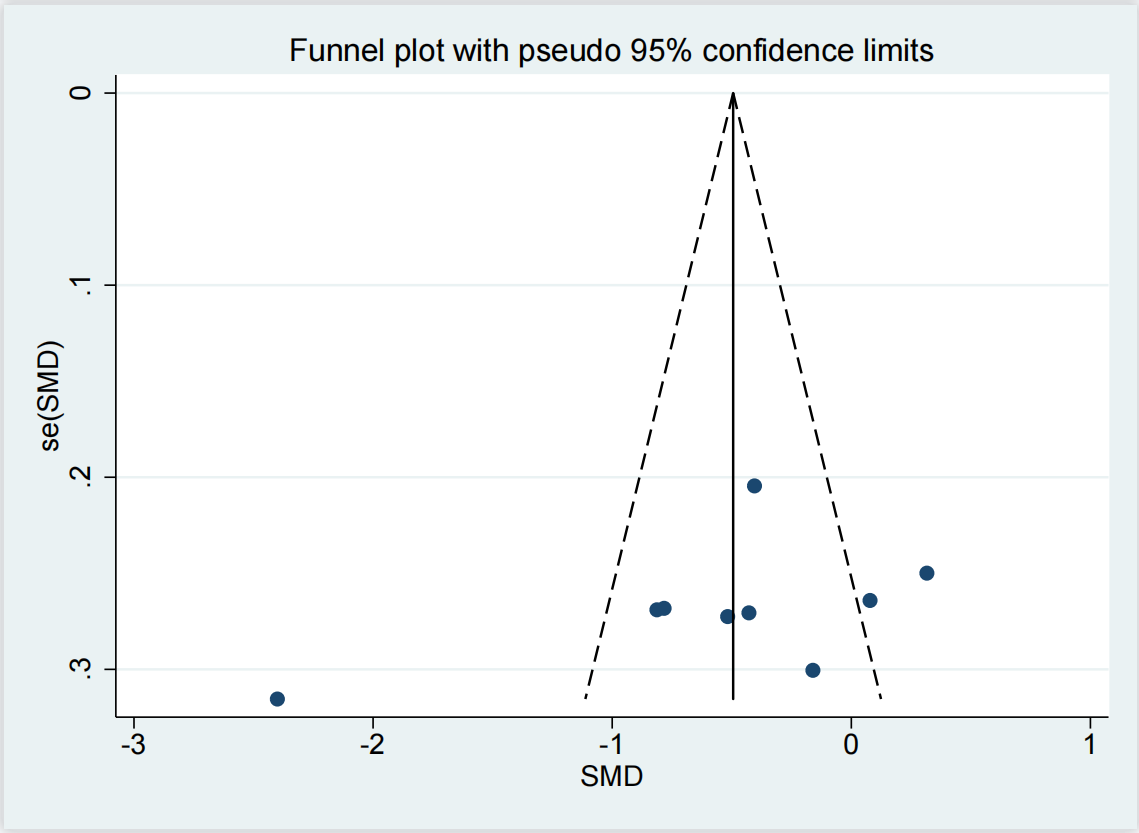


The abscissa is the effect size standard mean difference (SMD), the ordinate is the standard error of the effect size, the dots represent the studies included; funnel plot is symmetric, three dots fell outside the 95% confidence interval, Egger’s test *P* = 0.337.

**D: Funnel plot of Serum Leptin**


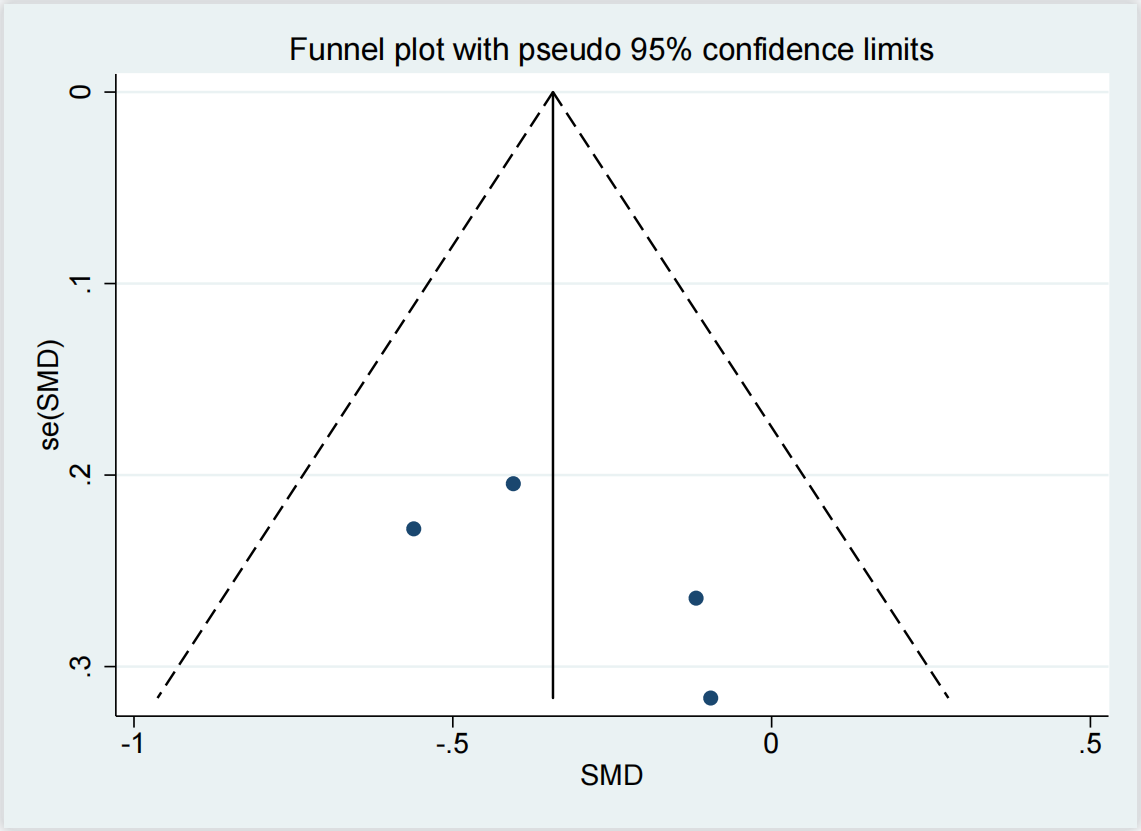


The abscissa is the effect size standard mean difference (SMD), the ordinate is the standard error of the effect size, the dots represent the studies included; funnel plot is symmetry, Egger’s test *P* = 0.095.

**E: Funnel plot of TC (total cholesterol)**


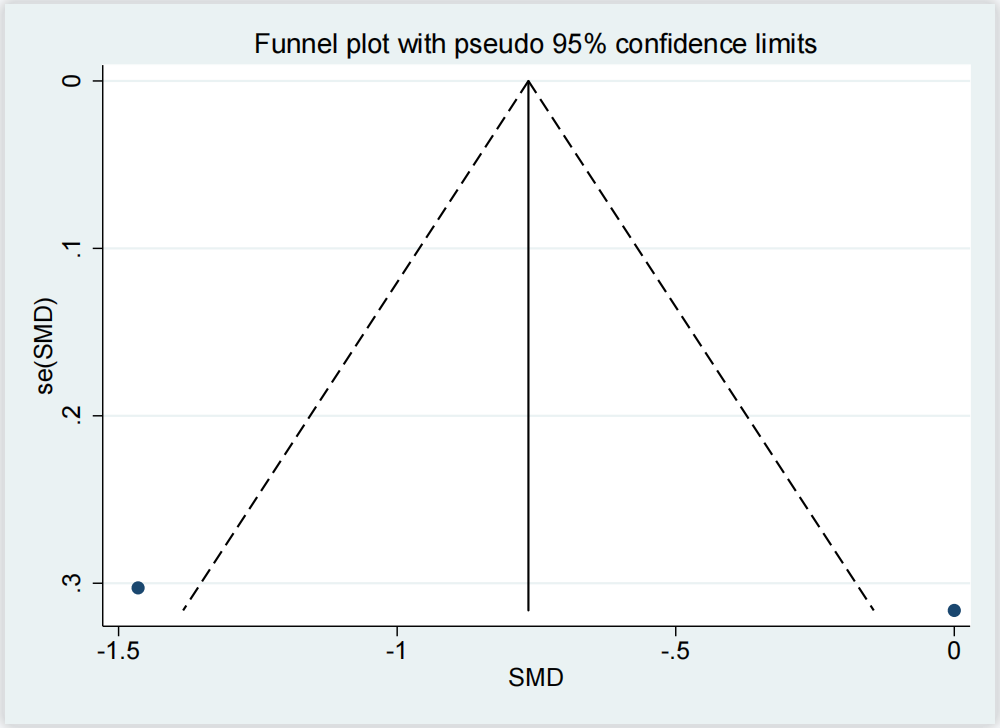


The abscissa is the effect size standard mean difference (SMD), the ordinate is the standard error of the effect size, the dots represent the studies included; funnel plot is symmetry, two dots fell outside the 95% confidence interval.

**F: Funnel plot of TG (triglyceride)**


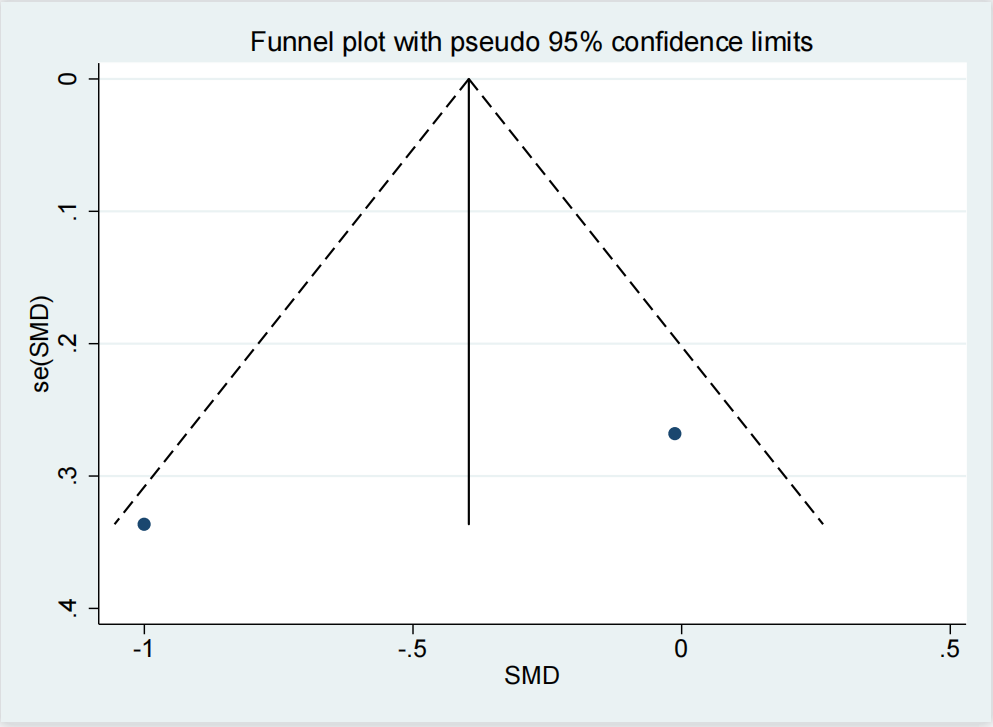


The abscissa is the effect size standard mean difference (SMD), the ordinate is the standard error of the effect size, the dots represent the studies included; funnel plot is symmetry.

**G:** **Funnel plot of LDL (low-density lipoprotein)**


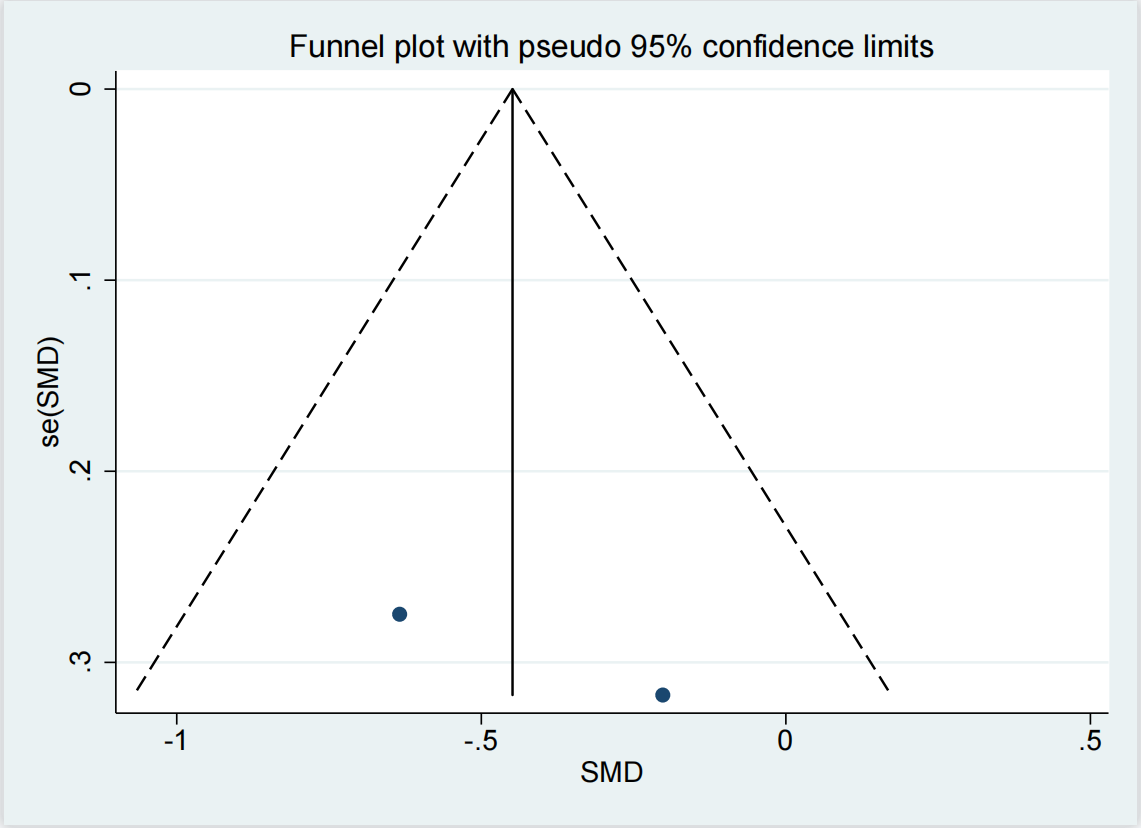


The abscissa is the effect size standard mean difference (SMD), the ordinate is the standard error of the effect size, the dots represent the studies included; funnel plot is symmetry.

**H: Funnel plot of HDL (high-density lipoprotein)**


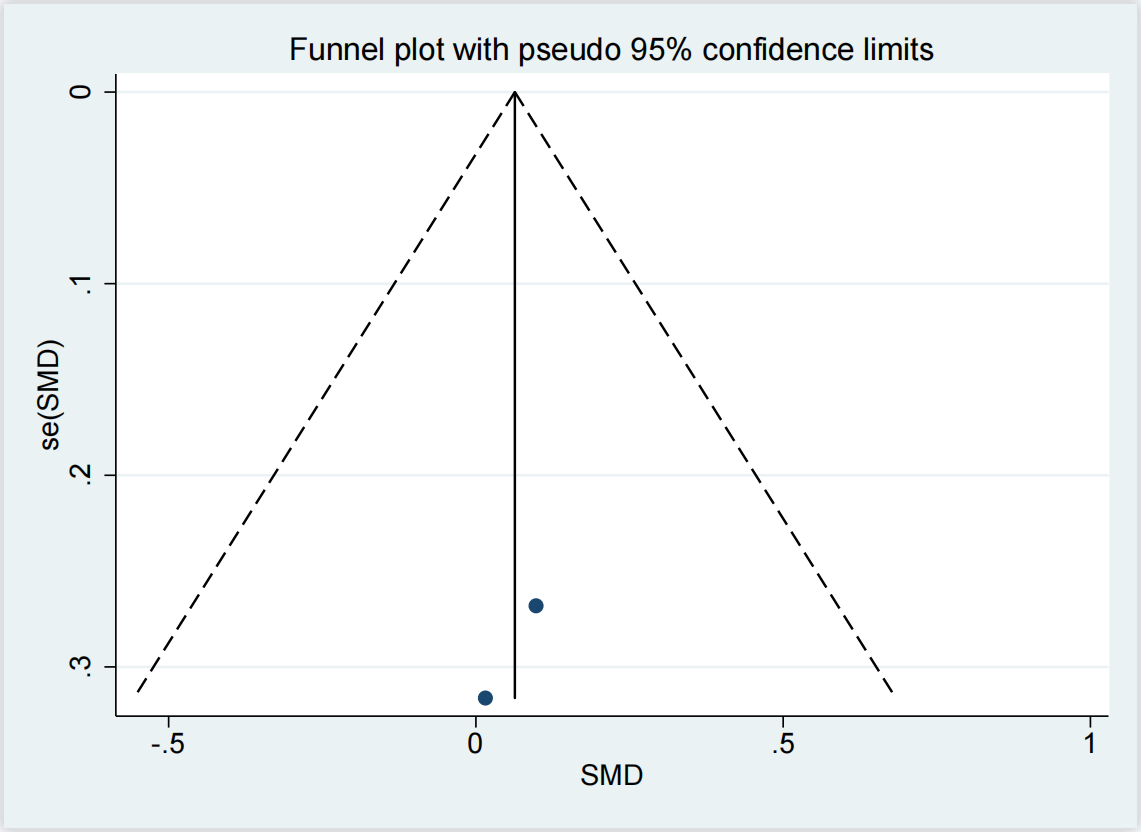


The abscissa is the effect size standard mean difference (SMD), the ordinate is the standard error of the effect size, the dots represent the studies included; funnel plot is symmetry.
